# Dental Hygiene

**Published:** 1869-08

**Authors:** Samuel Welchens


### ARTICLE VIII.

### *Dental Hygiene.*

By Samuel Welchens, D.D.S.

[Read before the Pennsylvania State Dental Society, at Harrisburg, June 8th, 1869.]

The importance of a good hygienic system in the several branches of medicine cannot be over-estimated. To preserve health should claim the attention of science, fully as much as the means of restoring it when impaired or lost. The physiological relations of the various parts of the human structure are so nicely balanced, while the elements of life and death are in such close proximity, striving continually for the mastery, that the highest attainment in professional skill, and the broadest and most comprehensive experience should be brought into requisition, in order to keep the phenomena of *vital activity* in channels of health and happiness. *Dental hygiene*, though to some extent a specialty, partakes in a very large degree of the elements of dependency. It involves some of the most important functions, and must, from the very nature of the case, be conditioned and influenced by the general laws of the economy.

The capillary network, which derives its nourishment from the systemic circulation, constitutes the source of vitality to the teeth, and to keep it in an active, healthy condition, so as to perform its functions properly, and build up and establish those hard unyielding structures, and develope, in the process of growth, a strong and enduring denture, is the subject of our present inquiry.

The human teeth are of that class of organs which are not in any direct relation with the blood-vessels, and yet which derive their whole nutriment, and the material of their functional operations, from the blood. In all other respects they are almost a separate and distinct creation. They begin to form, and become developed, when all the other organs are already faithful to the want of the economy, and are destroyed by disease and decay with but little serious inconvenience to the system.

While they are regarded as the hardest organic substance known, they are by no means the most durable. By their superior structure, they were evidently intended by nature to withstand all the wear and tear of mastication. But, apart from this, they are destined to sustain shocks and trials from internal derangements and external abuses, so that the office allotted to them is performed between two most powerful engines of destruction.

The rule seems to be, so far, at least, as external usage is concerned, neglect and violence ; and, where this is not directly the case, with regard to the teeth, their low vital powers are driven in upon themselves, from the surface, by some congealing agent, or raised to a state of inflammatory excitement by acids and corrosive applications, fully as destructive to their general health.

This kind of abuse is a fruitful cause of decay, but it obtains as much among those who are scrupulously cleanly, as those who are careless and filthy; so that the popular theory that the decay of those organs is caused solely and entirely by external agents is, for the most part, erroneous.

Our purpose, in this paper, is to combat this theory, and maintain that the cause of decay in the teeth is attributable to internal derangements and an insufficiency of capillary nutrition, fully as much, if not more, than to careless habits and the various causes of external abuse ; not, however, for the purpose of disparaging sanitary measures, but to direct, if possible, attention to the internal—so that our hygienic principles may apply and benefit the race from both standpoints and in both theories.

I hold that there are three several conditions of the economy, in which it is possible to so derange the functional powers as to superinduce a corresponding derangement of the capillary nutrition in tissues; and the complex organic nature and structure of the teeth being such as to deprive them of a rapid recuperative action, *stagnation*, leading to *decompositions and decays*, is the result.

The first of these conditions is that pathological state produced by mesenteric derangements and eruptive fevers incident to child-bed, whereby there is an undue consumption of capillary nutrition, and inability on the part of the tissues to appropriate proper aliment, which produces not only weak and delicate teeth, about the time of their formation, but the unsightly deformity of what is termed *dental atrophy*.

The second condition is an enfeebling or weakening process in the systemic circulation, by which the function of assimilation is retarded or defeated altogether, especially in the hard tissues of which the teeth are composed, and thus rendering them soft and liable to disease and decay.

The third condition or element of decay in dental tissue arises from an undue acceleration of the local circulation, producing active congestion in the soft tissues, a gorging or choking of the vesicles by a too rapid interstitial deposit of the mineral substances, designed by the economy to harden the teeth.

The foundation of a building or the fountain-head of a stream, must possess the ability to sustain that which depends upon it. The root of a plant must have absorbing surface to supply the wants of the stem and leaves. These are *axioms*, which will readily illustrate the absolute necessity of sufficient vital energy in the circulating system of the animal to supply a proper amount of nutriment to stimulate the entire capillary maze.

In all organic structures there are central or vital organs which supply functional power to those which are thrown to the surface, and the arrangement is to always have those organs deeply seated for their own protection, and that each succeeding strata of the fabric, as the growth is outward and upward, becomes more complex, and consequently less liable to injury, but at the same time less capable of recuperating their power when injured.

This general law of the economy is well-settled and most beautifully illustrated in the arrangement and formation of the human teeth.

The "*cementum*," or "*crusta petrosa*," the "*dentine*" and the "*enamel*" constitute the three several divisions of the dental tissue, and the arrangement gives the *cementum* the position nearest the vascular tissue, because of its better adaptation to hold the fluid thrown in from the blood to be imbibed by the capillaries of the more highly organized structure of the *dentine*. And then, again, the *enamel* being still more dense, and less liable to injury by reason of its low vital organization, comes boldly to the surface, and finishes the grandest structure of the animal economy.

The cementum contains about one-third per centum of animal matter, thus rendering its "*lacunæ*," and "*canaliculi*" capable of retaining, and, to some extent, elaborating, the fluid which is secreted from the *periosteum* to nourish the *dentine*, by the process of *endosmosis*, or *imbibition*. This structure, then, is that which holds the destiny of the entire tooth. The ability of the organism to keep the *dentine* in a healthy state, by preserving a proper vital balance, is the very pith of our subject.

The dentine is, perhaps, the most delicately organized fabric within the entire range of the economy. Its capillary vesicles contain a fluid composed of the finest particles of the blood. This fluid is laden with the azotized compounds designed by nature to build up the tissue, and by virtue of its extremely delicate and sensitive character, it is easily influenced, either to a healthy, vigorous action, by a proper functional stimulant from the systemic circulation, or it can be disorganized, either from the want of such a stimulant, or a too rapid interstitial deposit of its particles. These changes can take place through disease, a feeble organization, or external abuses.

In marasmus, catarrah fever, measles, or any of those diseases incident to childhood, the integrity of the blood becomes destroyed, capillary circulation and nutrition are interrupted and deranged. And when they are contracted about the time of the formation of the dental tissue, there is an insufficiency of mineral matter for a perfect development of the teeth, and especially of the enamel, and the result is that hideous malformation termed "*dental atrophy.*" Or a lack of *vital energy* may be superinduced from the same cause, and though there may be material enough to form a beautiful pearly denture, the organization does not go on vigorous enough to make it strong and enduring, and a soft chalky *enamel*, with a corresponing enfeebled *dentine*, is the result.

Sometimes, too, those pathological conditions are entailed from parent to child. This consumption of vital energy, and of capillary nutrition, thus become congenital, and that which is lost by the parent can never be regained by the child. The teeth always become involved in those defections, and can never be rendered strong and enduring in texture.

These are pathological conditions of the system, many of which must be allowed to run their course, and do their work of destruction. All the dentist can do is to apply his artistic skill, either in treating the natural organ, or supplying an artificial denture—there being no system of dental hygiene fully equal to the task.

We will therefore turn to our next element of inquiry.

An insufficiency of capillary nutrition, from whatever cause, will have a more damaging effect upon the teeth than any other part of the capillary system. A derangement of this sort in the epithelial and epidermic, and also the articular catilage vessels, can be more rapidly overcome, by reason of the flexibility of their walls, and their adaptation to the laws of tolerance prevading the entire structure. But the denser and more highly organized fabric which composes the teeth, is not possessed of such rallying power, and consequently must suffer injury corresponding to the amount of damage to the other tissues, done by such irregularity; and, not being able to recover as rapidly as the softer tissues, the the injury multiplies—disintegration is followed by disorganization and decay.

The operation of nature in the vegetable kingdom is analogous to this, and will serve to illustrate the idea we wish to present. So long as the root of a tree promptly answers the demands of the trunk and leaves, all goes well; but when this supply fails, the foliage begins to flag and droop, and if this exhaustion proceeds beyond a certain point, the leaves wither and perish. If there is a deficiency or an irregularity of the absorbing surface of the root, it may not destroy the entire foliage, but, "leaf by leaf it will droop and die." The teeth *can decay*, and in a majority of cases *do decay*, from similar circumstances.

The cause, in every instance of this deficiency of "*capillary nutrition*," cannot be discovered. It occurs in all grades and ages of the human race, and does not escape the robust and healthy any more than those who are constitutionally delicate.

Irregularities in the capillary circulation have been noted when the systemic circulation was in perfect condition. They are most frequent, however, when the heart's action is enfeebled or partially interrupted. And this can occur, and most generally does occur, through some of the abuses of the system incident to our modern mode of life. Dissipation, exposure, sudden and extreme changes in temperature and habits, improper and insufficient diet, tight lacing, careless and filthy living and customs, are among the primary and principal causes. Each and all of these irregular habits of life may produce great and sudden variations, either in whole or part of the capillary network—the circulation taking place with diminished rapidity in one part, and increased energy in another, though both are supplied by the same trunk. These are not the results of a chronic pathological condition of functional power, but a sequence of their neglect and abuse.

Go into the candy shops, the fashionable hotels and boarding houses, and see the amazing consumption of dainties, well calculated to disturb the normal condition of the system, and, instead of being aliment for a healthy development of the tissues, they produce those debilitating results. Go into the festive social parties, and the brilliant entertainments and balls where so many of the young, particularly in our large cities, almost live in seasons of gaiety and fashion—where the action of the larger organs is destroyed, and the functional power of systemic circulation is retarded, and the vital energy of the system is driven back upon its centre by the half sufficient dress, the congealing efficacy of a piercing atmosphere, and unseasonable hours peculiar to such a life. Go into the haunts of vice and dissipation, and mark the manner in which this grand human structure is abused by those whose interest it is to preserve it, and you have a solution at once of the problem we are endeavoring to solve.

But come home, and see whether your little son or little daughter is not suffering the same loss of capillary nutrition and functional energy by close confinement, either in the house or at school, or by the use of too many candies, too much ice cream, or by being over-fed at table with too much sweetened bread and a corresponding round of rich pastry and dainty dessert, without a balancing supply of the more solid food. Here it is that "*dental hygiene*" should interpose its gracious offices, to regulate these dissolute habits. Insist upon proper treatment in the case of children, and thus re-establish that measure of vital energy which our race is rapidly losing, by not knowing exactly how to live.

We may, however, be pointed to a class of people whose habits in life preclude the idea of their participation in any of those scenes of exposure and dissipation, or whose children are rarely indulged as above intimated, but where there seems to be fully as much decay in the dental tissues. Look at our sturdy farmers and woodsmen, and suburban laborers and mechanics throughout the country. They and their children are, for the most part, strangers to those excesses, and seem to be the very embodiment of robust health and manly development, and yet they come into our offices with as much dental distress as those above enumerated.

With this class of people *sanitary measures* will go far, very far, towards a propitiation of dental decay, and in perhaps the majority of cases will prevent disease until there is a proper development and enduring texture produced in the tissues. But, with all the care that can be taken, all along through life, at every stage and period, they are confronted with the stern reality of an *exposed* and *aching pulp*, through the decay and breaking in of the crowns or walls of the teeth. Can all this be said to be the result of external agencies and injuries *alone?* May there not, after all, be some cause of a character purely and entirely *internal* from which such results may obtain?

We answer, that we believe that there is, and the theory which we hold on the subject will claim the principles we design to discuss in our third and last proposition, namely: the liability of teeth to decay through an undue acceleration of local or capillary circulation, from which there is a gorging or choking of the tissues, thereby interrupting functional power, and producing results similar to an enfeebling insufficiency of capillary nutrition.

In the tree, in the vegetable kingdom, for example, in seasons of active vegetation, leaves may be observed to wither, die and fall off, notwithstanding the fact that the foliage seems strong and vigorous. This is the result of an undue acceleration of the circulation of the sap. Those leaves become gorged and choked by a too rapid deposit of the saline and earthly particles designed by nature to build up the tissue, and their destruction is the result of this arrest of functional activity.

"The blood, when circulating through the *systemic* capillaries, yields a portion of its oxygen to the tissues it permeates, and receives from them carbonic acid. On the other hand, when passing through the *pulmonary* capillaries, it gives up its carbonic acid to the atmosphere, and imbibes a fresh supply of oxygen. Now, if either of these changes be prevented from taking place, a retardation, and even a complete stagnation of the blood will take place, the flow through the capillaries being now resisted, instead of accelerated, by the relation which the blood bears to the tissues."

"The change in the condition of the blood, in regard to the relative proportions of its oxygen and carbonic acid, is the only one to which the pulmonary circulation is subservient; but in the systemic circulation, the changes are of a much more complex nature—every distinct organ attracting to itself the peculiar substances which it requires as the materials of its own nutrition." An acceleration when undue and abnormal, of this character, is known as "*active congestion*," or "*determination of blood*." In the softer tissues these pathological conditions can be arrested before inflammation or stagnation sets in; but in the denser or bony tissues, and especially those forming the teeth, the remedy cannot be applied; the interstitial deposit cannot be corrected or reduced to a normal standard, and a stagnation is the result, which finally produces disorganization and decay.

These changes in the capillary circulation may not indicate distress in any of the larger organs, or their functions. The system may be entirely free from any diseased condition of the vital forces. But as the individual, standing upon the shore of a river, may drink in miasmatic poison from the grateful breeze that fans his over-heated and sweated brow, so may those hard unyielding organs receive the seeds of decay and death in the very flow of life and spirits which bring the glow of health and beauty upon the cheek by the extending walls of the capillary maze, which allow the red corpuscles of the blood to come to the surface by reason of an acceleration of the circulation.

It is rarely, indeed, that the diseases of the teeth can be traced to such delightful surroundings, or such fine robust conditions. But is the fact not apparent, at every step of scientific research, that the elements of life and death are not only running side by side continually thorough our veins and arteries, but that every step in life, and every phase of enjoyment, are beset with dangers which sometimes suddenly lead to the grave.

Here "*dental hygiene*" is at fault. The dentist, however skillful and thoroughly scientific, must stand abashed before these freaks and mandates of nature, and ply his skill in operations upon the diseased organ, or allow the work of decay and death to run its course, and restore the function by an artificial denture. These results do not flow from the same causes, before enumerated, and consequently cannot be met by the same hygienic treatment; but they confront us at every step of our experience in active practice, and, therefore, demand our attention.

Perhaps the best and simplest definition of the idea of "*dental hygiene*" may be found in the phraseology, "*raise the teeth*," as you would raise a child or a tender plant.

Observe all the rules known to what might be termed a *sanitary regime*, and you meet the demands of the economy so far as external causes and agencies are concerned. This treatment goes very far toward a good development of the dental organs; but, as we maintain that the cause of decay is not at all times of this outward character, this kind of treatment *alone* is not sufficient. To raise a child well, the process of ablution is not all that is required. There are laws and principles, the observance of which is not only necessary to a healthy growth of the system, but to regulate the habits, the diet and appetite, and a good mental and moral development of the faculties are all necessary. Good substantial food, containing all the elements necessary to build up and nourish the various tissues of the body—clean, warm clothing to protect the surface from the chilling blasts of winter, and regular out-door exercise—all, with temperance and moderation, will not only raise the child well, but, in a large majority of cases, *raise a denture* well calculated to withstand the changes of life, and endure the wear and tear of mastication.

We cannot crowd all the out-croppings and freaks of nature into an easy subserviency to hygienic laws; but in the main, with the deeper intuitions of observant and well-balanced minds, every community can be so educated to general principles as to aid, through popular sentiment, an improvement in the general physiological status of the race.

All seem to comprehend the art and mystery of destroying the human fabric. They seem to know what steps to take to run this magnificent structure into decay and death, but are slow—very slow—to grasp the idea of health and happiness.

The simplest way to raise a child well, *is to desist from abusing it.* Break in at once and forever upon those habits and fashions which are leading children, almost from lisping infancy, into the maelstrom of extravagance and dissipation. Resist the overpowering pressure of the baser and more vulgar customs of modern life, and yield to the surer and better dictates of nature, (if there is no other help at hand), and the work is done.

But, to raise a *regular*, *enduring denture*, is not always the work of unaided nature. A careful harmonizing of the conditions of growth with the principles of science and art, is the prerogative of our profession, and which should be exercised whenever there seems to be an inability in nature to perform its task.

In the finest and best developed forms the teeth often present themselves in most unsightly irregularities. In such cases the skill of the dentist must be brought into requisition, for, however strong in texture such teeth may be, their crowded condition will render them liable to disease from external causes.

A few thoughts in regard to the liability of teeth to decay from neglect and abuse, and the influence of acids and alkalies as they come up, either in the secretions of the mouth, in food, or by direct contact, and we are done.

The low vital power of the enamel renders it susceptible to great and lasting injury, where any of the last named reagents come in direct contact with it. It contains so small a per centum of animal matter that those strong poisonous substances destroy the vital principle, and leave the mineral structure a dead, disorganized mass of matter, which breaks away in the process of mastication, or shows signs of disintegration in black, decayed spots, or a discoloration of the enamel altogether. These evidences of decay often present themselves where no such corrosive action can take place; but the whole theory of external injury must rest upon the hypothesis of some such decomposing agent, constituting, either directly or in an indirect manner, the disease of this hardest of all organized bodies.

That a powerful corrosive action can take place by the decomposing of animal matter, or vegetable subtances, through the action of the saliva, when such substances are allowed to remain upon or between the teeth, no one will undertake to deny; and that many teeth are destroyed from such filthy neglect is also a fact beyond controversy. But in most cases, even when those elements are present, and where those more active and more deadly reagents, before mentioned, do come in contact, the influence they exert is very much over-estimated.

If the enamel is of a first-class development, and there are none of the internal causes above enumerated present, such neglect or abuse will be very slow in producing an entire destruction of the denture, or even the decay of an occasional tooth. Where is the practitioner of half a dozen year's experience who has not seen beautiful dentures, of that iron texture, without spot or blemish, save a slight abrasion of the crown, where the possessor can boast of his ability to "*clip in two*" a ten-penny nail? And this, too, with the other boast of never having touched their teeth with a brush. The Irishman and the German often present such developments, but they are rarely seen in the more modern circles of American life.

We, as Americans, are, however, not without beautiful specimens of first-class teeth, which, too, can be, and in the majority of cases are, preserved through care without a spot of caries, until a late period of life, when, by a sloughing of the "*periosteum*," or an absorption of the integuments, they loosen, become painful, and must be removed.

Much, too, has been said and written about "*acids*" and "*alkalies*," as they come up in the food and condiments in use by modern epicures. Those deductions and conclusions, we think, are strained and largely over-estimated. When mixed and baked, and boiled and cured, they come in contact with the teeth as entirely different chemical compounds, and their influence is no longer acid nor alkaline, but so mild in their effects, especially regarding the fact that they are held there so short a time, that very little injury can be caused by them.

Much of the distress, however, occasioned by all the above enumerated agents, can be prevented by a proper system of hygienic treatment. In order properly to meet all the contingencies necessary, and to comply with the suggestions of science, in the work of *raising* and *preserving* a good, enduring and substantial denture, the dental practitioner should have control of the process of dentition from infancy, and much of the manner of the child's living. The eruption of those organs should be an operation of special care, and when they are fairly developed, frequent inspection and good sanitary directions, with all the treatment, such as the nature of the case may require, and you meet, in a scientific way, all the mandates of what we understand by *dental hygiene.—Dental Times.*

